# Infectious mononucleosis is associated with an increased incidence of multiple sclerosis: Results from a cohort study of 32,116 outpatients in Germany

**DOI:** 10.3389/fimmu.2022.937583

**Published:** 2022-08-02

**Authors:** Sven H. Loosen, Corinna Doege, Sven G. Meuth, Tom Luedde, Karel Kostev, Christoph Roderburg

**Affiliations:** ^1^ Clinic for Gastroenterology, Hepatology and Infectious Diseases, University Hospital Düsseldorf, Medical Faculty of Heinrich Heine University Düsseldorf, Düsseldorf, Germany; ^2^ Department of Pediatric Neurology, Center of Pediatrics and Adolescent Medicine, Central Hospital Bremen, Bremen, Germany; ^3^ Department of Neurology, Medical Faculty, University Hospital, Heinrich-Heine-University, Düsseldorf, Germany; ^4^ Epidemiology, IQVIA, Frankfurt, Germany

**Keywords:** EBV, MS, pathophysiology, virus, incidence, epidemiology

## Abstract

**Background:**

The pathogenesis of multiple sclerosis (MS) has not yet been fully uncovered. There is increasing evidence that Epstein-Barr-Virus (EBV) infection, which affects over 90% of people during life and causes infectious mononucleosis, leads to an increased incidence of MS, and thus may play a crucial role in the pathophysiology of the disease.

**Methods:**

Using the Disease Analyzer database (IQVIA) featuring diagnoses as well as basic medical and demographic data of outpatients from general practices in Germany, we identified a total of 16,058 patients with infectious mononucleosis that were matched to a cohort of equal size without infectious mononucleosis based on patients’ age, sex, index year and yearly consultation frequency. Incidence of MS was compared within a 10-year follow-up period.

**Results:**

Within 10 years from the index date, the incidence of MS was 22.6 cases per 100,000 person-years among patient with infectious mononucleosis but only 11.9 cases per 100,000 person-years among individuals without infectious mononucleosis. In regression analysis, infectious mononucleosis was significantly associated with the incidence of MS (HR: 1.86, 95% CI: 1.09-3.16). Subgroup analysis revealed the strongest association between infectious mononucleosis and MS in the age group between 14 and 20 years (HR: 3.52, 95% CI: 1.00-12.37) as well as a stronger association in men compared to women.

**Conclusion:**

Infectious mononucleosis is associated with an increased incidence of MS especially in younger individuals. Our data support the growing evidence of a decisive involvement of EBV in the currently unknown pathophysiology of MS and should trigger further research efforts to better understand and potentially prevent cases of this disabling disease in future.

## Introduction

Multiple sclerosis (MS) is an autoimmune disease of the central nervous system that causes immune-mediated demyelination and axonal degeneration, resulting in progressive neurological disability that affects over two million people globally (1, 2). MS is characterized by symptoms of neurological disability such as limb weakness or sensory loss, monocular visual loss, double vision or ataxia that occur periodically over a period from days to weeks and are fully or partially reversible (3). As the majority of patients eventually face a progressive clinical course leading to a decisively impaired mobility and cognition, MS is associated with a tremendous personal as well as socioeconomic burden (2). Currently, there is no approved treatment that can fully prevent or even reverse the progressive neurological deterioration (2). Therefore, a definite understanding of the pathophysiology of MS, which has not been elucidated so far, is of crucial importance to establish potential preventive measures.

Recently, the previously suspected hypothesis of a possible link between EBV infection, which affects over 90% of the population in Western countries during life and causes infectious mononucleosis, and the development of MS has been confirmed in a large collective of over 10 million young adults from the United States (4). The risk of MS increased up to 32-fold in individuals infected with EBV but not other viruses, including cytomegalovirus (4). Moreover, a causal relationship between EBV and MS is supported by data on elevated antibody titers against EBV nuclear antigens in MS patients (5) as well as the presence of EBV in demyelinated MS lesions (6, 7). However, evidence of a direct causality is still discussed controversially (8).

In order to further dissect the association between EBV infection and the incidence of MS, we performed a retrospective cohort study based on a large sample of outpatients in Germany. These data may help to further elucidate the pathophysiological involvement of EBV in the development of MS and to further advance possible preventive measures such as the development of a vaccine against EBV.

## Materials and methods

### Database

This present study uses the Disease Analyzer database (IQVIA), which contains drug prescriptions, diagnoses as well as basic medical and demographic data that are directly obtained in an anonymous format from general practitioners (9). The database covers approximately 3% of all outpatient practices in Germany. Diagnoses (according to International Classification of Diseases, 10th revision [ICD-10]), prescriptions (according to Anatomical Therapeutic Chemical [ATC] Classification system), and the quality of reported data are being monitored by IQVIA. In Germany, the sampling methods used to select physicians’ practices are appropriate for obtaining a representative database of general and specialized practices. It has previously been shown that the panel of practices included in the Disease Analyzer database is representative of general and specialized practices in Germany (9). Finally, this database has already been used in previous studies focusing on infectious mononucleosis (10) as well as MS (11, 12).

### Study population

**Figure 1:**
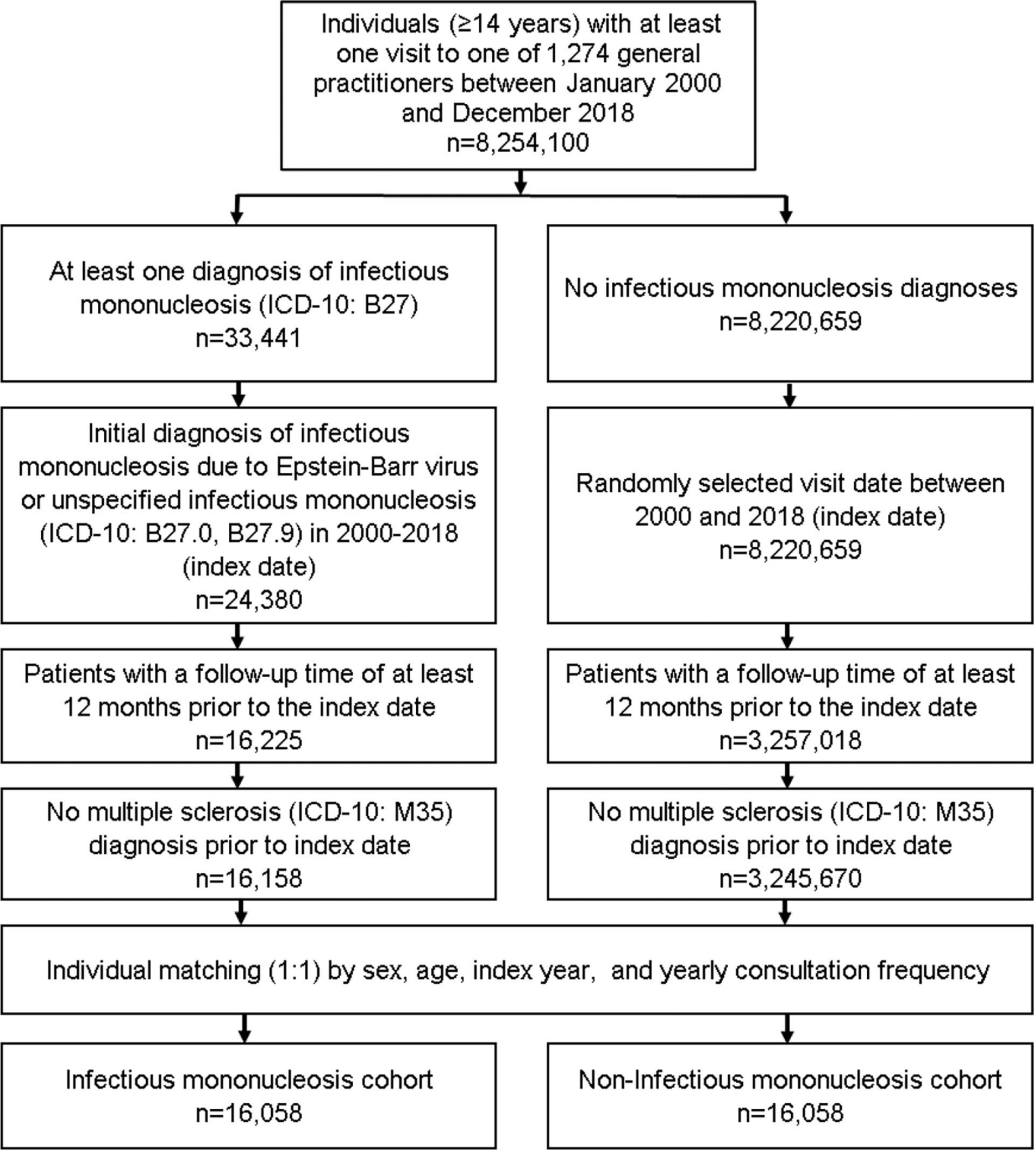
Selection of study patients.

This retrospective cohort study included patients (≥14 years) with an initial diagnosis of infectious mononucleosis (ICD-10: B27) in 1,274 general practices in Germany between January 2000 and December 2018 (index date; **Figure 1**). Further inclusion criterion was an observation time of at least 12 months prior to the index date. Patients with a diagnosis of MS (ICD-10: M35) prior to index date were excluded. Patients with infectious mononucleosis were matched to individuals without infectious mononucleosis by sex, age, index year, and yearly consultation frequency. As infectious mononucleosis patients have a much higher consultation frequency at their GPs, and a higher consultation frequency can increase the probability of other diagnoses documentation, we also included consultation frequency per year in the matching process. For the individuals without infectious mononucleosis, the index date was that of a randomly selected visit between January 2000 and December 2018 (**Figure 1**).

### Study outcomes and statistical analyses

The main outcome of the study was the incidence of MS (ICD-10: M35) as a function of infectious mononucleosis. Differences in the sample characteristics between individuals with and without infectious mononucleosis were tested using chi-squared tests for categorical variables and Wilcoxon tests for continuous variables. Univariable Cox regression models *were conducted to study the association between infectious mononucleosis and the incidence* of MS. These models were performed separately for women, men and five age groups. Finally, multivariable Cox regression models were conducted, adjusted for six autoimmune disorders including type 1 diabetes (ICD-10: E10), rheumatoid arthritis (ICD-10: M05, M06), psoriasis (ICD-10: L40), lupus erythematosus (ICD-10: M32), Hashimoto’s thyroiditis (ICD-10: E06.3), inflammatory bowel disease (ICD-10: K50, K51). P-values <0.05 were considered statistically significant. Analyses were carried out using SAS version 9.4 (SAS institute, Cary, USA).

## Results

### Characteristics of the study cohort

The present study included 16,058 patients with documented infectious mononucleosis as well as 16,058 matched individuals without infectious mononucleosis. The basic characteristics of the study cohort are summarized in **Table 1**. The mean age (SD) was 32 years (15 years), whereby 47% of patients were between 14 and 30 years old. 58.6% of patients were female. 5.7% of individuals with and 5.6% of individuals without infectious mononucleosis were diagnosed with at least one pre-defined autoimmune disorders at baseline. On average, patients visited their general practitioner 3.5 times per year during the follow-up period.

**Table 1:** Basic characteristics of the study sample (after 1:1 matching by sex, age, index year, and yearly consultation frequency).

Variable	Proportion affected among individuals with infectious mononucleosis (%)	Proportion affected among individuals without infectious mononucleosis (%)	p-value
	n=16,058	n=16,058	
Age (Mean, SD)	31.6 (15.0)	31.7 (15.1)	0.494
Age 14-20	4,969 (30.9)	4,815 (30.0)	0.440
Age 21-30	4,240 (26.4)	4,325 (26.9)	
Age 31-40	2,734 (17.0)	2,740 (17.1)	
Age 41-50	1,991 (12.4)	2,024 (12.6)	
Age >50	2,124 (13.2)	2,154 (13.4)	
Women	9,407 (58.6)	9,407 (58.6)	1.000
Men	6,651 (41.4)	6,651 (41.4)	
Yearly consultation frequency	3.5 (4.3)	3.5 (4.3)	1.000
Autoimmune disorders (any)	908 (5.7)	892 (5.6)	0.700
Type 1 diabetes	60 (0.4)	129 (0.8)	<0.001
Rheumatoid arthritis	228 (1.4)	164 (1.0)	0.001
Psoriasis	226 (1.4)	223 (1.4)	0.887
Lupus erythematosus	7 (0.04)	3 (0.02)	0.206
Hashimoto’s thyroiditis	332 (2.1)	294 (1.8)	0.125
Inflammatory bowel disease	118 (0.7)	125 (0.8)	0.652

Proportions of patients given in %, unless otherwise indicated. SD, standard deviation.

### Association of infectious mononucleosis and the incidence of multiple sclerosis

**Figure 2:**
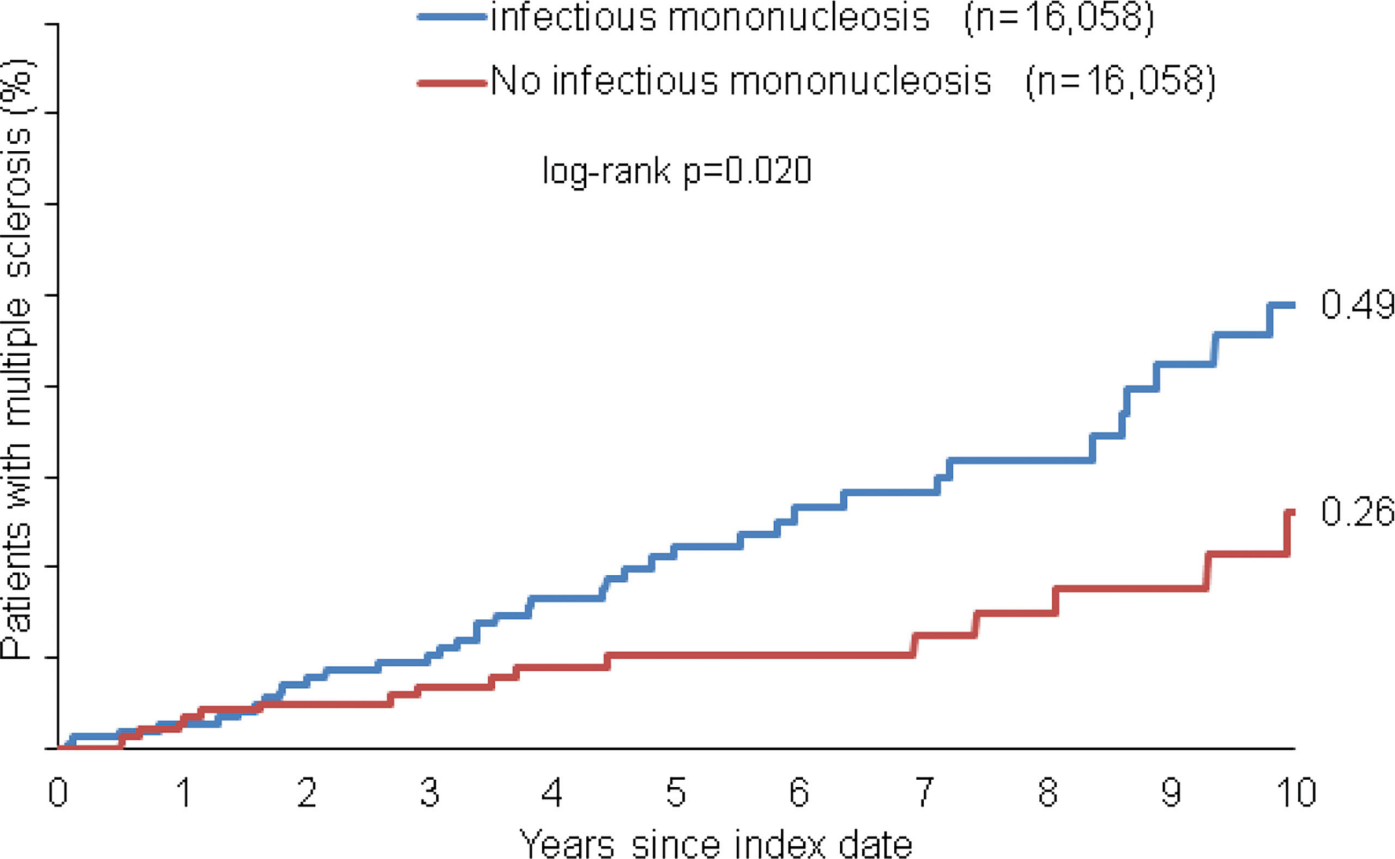
Kaplan-Meier curves regarding the incidence of multiple sclerosis diagnosis in patients with and without infectious mononucleosis.

Within 10 years from the index date, a total of 0.49% of patients with infectious mononucleosis and 0.26% of individuals without infectious mononucleosis were diagnosed with multiple sclerosis (MS, log-rank: p<0.001, **Figure 2**). The incidence of MS was 22.6 cases per 100,000 person-years among patients with infectious mononucleosis and 11.9 cases per 100,000 person-years among individuals without infectious mononucleosis.

In univariable regression analyses, infectious mononucleosis was significantly associated with the incidence of MS (HR: 1.86, 95% CI: 1.09-3.16, **Table 2**). In age-stratified analysis however, the only age group with significant association was 14-20 years (HR: 3.52, 95% CI: 1.00-12.37). With increased age, the association between infectious mononucleosis and MS became smaller and was not significant. The association in men was stronger than in women, however both values were not significant due to small samples (**Table 2**). Similar results were observed following adjustment for pre-existing autoimmune disorders (see materials and methods for details) in a multivariable regression model (**Table 2**).

**Table 2:** Association between infectious mononucleosis and the subsequent incident multiple sclerosis diagnosis in patients followed in general practices in Germany (Cox regression models).

			Univariable regression		Multivariable regression*	
Cohorts	Incidence of MS among individuals with infectious mononucleosis (cases per 100,000 patient years)	Incidence of MS among individuals without infectious mononucleosis (cases per 100,000 patient years)	Hazard Ratios (95% CI)	p-values	Hazard Ratios (95% CI)	p-values
Total	22.6	11.9	1.86 (1.09-3.16)	0.022	1.88 (1.10-3.19)	0.021
Age 14-20	22.6	6.1	3.52 (1.00-12.37)	0.049	4.03 (1.12-14.52)	0.033
Age 21-30	29.6	17.5	1.74 (0.70-4.37)	0.236	1.73 (0.69-4.35)	0.241
Age 31-40	28.9	16.5	1.63 (0.55-4.77)	0.377	1.61 (0.55-4.72)	0.387
Age 41-50	14.9	12.2	1.25 (0.28-5.59)	0.771	1.22 (0.27-5.48)	0.793
Age >50	10.9	8.6	1.32 (0.22-7.92)	0.761	1.32 (0.22-7.94)	0.760
Women	27.1	15.2	1.72 (0.93-3.20)	0.086	1.76 (0.94-3.28)	0.076
Men	16.3	7.3	2.30 (0.82-6.46)	0.113	2.31 (0.82-6.48)	0.113

*Adjusted for autoimmune disorders (type 1 diabetes, rheumatoid arthritis, psoriasis, lupus erythematosus, Hashimoto’s thyroiditis, inflammatory bowel disease.

## Discussion

In this study, we investigated a potential association between infectious mononucleosis and the incidence of multiple sclerosis (MS). Therefore, we used data from a large outpatient database which collects diagnoses as well as basic medical and demographic data directly from general practitioners and specialists in Germany (9). Our results demonstrate that the incidence of MS is significantly higher in patients with infectious mononucleosis than in matched patients without infectious mononucleosis.

A possible causal relationship between infections with the Epstein-Barr virus and MS has been postulated before (5). One difficulty in investigating a possible association between infectious mononucleosis and MS is that, despite showing incidence rates of up to 1 of 200 population in high prevalence areas, the prevalence of MS is only about 0.1% in the general population (2), whereas EBV is ubiquitous, affecting up to 95% of all adults (13). A recently published study by Kjetil Bjornevik and colleagues, that inspired our study, solved this problem by observing over 10 million individuals serving as military personnel over an extended period of time. Of these, 5.3% were EBV negative and could be screened longitudinally for risk of EBV infection and development of MS (4). The authors showed that the risk of developing MS was significantly increased after infection with EBV, but not after infection with other viruses, such as cytomegalovirus, which is transmitted similarly to EBV.

Our own data confirm this important relationship in a German collective; in some respects, of course, our study differs from that of Bjornevik and co-workers. While we also followed up patients with documented EBV infection longitudinally over a period of 10 years and document the manifestation of MS, our data are not based on the analysis of serum samples but on the documentation of ICD-10 codes by primary care physicians. Of note, we can provide an analysis of relative risk across a wide range of different age groups. This analysis seems important from our point of view, since both infectious mononucleosis and MS have an age-dependent incidence pattern: MS has an incidence peak around the age of 30, but significantly earlier initial manifestations are also possible in childhood (14). The prevalence of EBV infection/seroconversion is already 50% in 6-8-year-olds and shows a steep increase to almost 90% in 18-19 year olds (15). With the onset of puberty, the proportion of EBV primary infection presenting as infectious mononucleosis increases markedly (16). The strongest association between infectious mononucleosis and MS was found in the youngest age group of our study, 14 to 20 years. There seems to be a particular risk here. This is all the more remarkable as the time course between the initiation of the MS disease process and the clinical manifestation is probably protracted (2), a time lag of 3.8 to 5.6 years is postulated between seroconversion and MS onset (17). Thus, to the extent that there is a causal relationship between EBV infection and MS incidence, it appears to be particularly effective at a young age. Conversely, it could be assumed that other risk factors for the occurrence of MS are pronounced in advanced age. Various genetic and environmental risk factors have been demonstrated: For reasons unknown so far, 3/4 of all affected are female (18). A genetic predisposition has been demonstrated, over 200 gene variants have been shown to be associated with an increased risk of developing MS of which the most prominent example is the human leukocyte antigen DRB1*1501 haplotype (odds ratio~3) (2). Environmental factors include climate zone (higher incidence in temperate latitudes), nicotine use and obesity.

Particularly through the study by Bjornevik et al. (4) as well as other studies, there is now a body of evidence that EBV infection potentially contributes to a breakdown of immune tolerance networks supporting the development of MS. For example, Ruprecht and co-workers observed that virtually all MS patients are EBV seropositive (19). This was also shown in a large German cohort of patients with early-stage MS (20). Prospective long-term studies found that high IgG titers directed against Epstein-Barr Nuclear Antigen (EBNA)-1 or EBNA complex (EBNAc) were associated with an increased risk of subsequent MS (5). For persons with an anti-EBNAc-Titer ≥ 320 the risk for developing MS was reported to be 30-fold in patients with EBNA complex IgG titers >/=320 than among those with titers <20 (21). Overall, our data reinforce previous research findings that EBV plays a causal role in the pathophysiology of MS. Under certain pathophysiological conditions, EBV seems to trigger specific biological changes that are required for the development of MS. However, the exact mechanism as well as the role of possible cofactors are still unclear.

An established concept that is discussed as a pathophysiological basis in various autoimmune diseases is that of molecular mimicry. This concept is based on a cellular or humoral immune response directed against infectious agents, which is misdirected by reacting with autoantigens (22). Another theory is that EBV may persist latently in autoreactive B lymphocytes. These in turn could induce autoreactive T cells as antigen-presenting cells, which could then ultimately trigger the organ damage in autoimmune diseases like MS (23). One of the arguments in favour of this theory is the close link between EBV replication and B cell biology (24). A recent study by Lanz et al. (25) combines both theories providing a mechanistic link between EBV infection and the pathobiology of MS: the authors extracted a cerebrospinal fluid (CSF)-derived mAb targeting EBNA1AA386-405 that reveals molecular mimicry to GlialCAM. This cross-reactivity represents a broader phenomenon in MS patients, as plasma blasts from the CSF of MS patients generate antibodies against multiple GliaICAM epitopes. The authors concluded that this mechanism could even represent a potential approach for innovative therapies for MS.

An important question is whether these associations and pathomechanisms are EBV-specific. In particular, herpes viruses such as cytomegalovirus (CMV) have a similar pattern of infection and contamination as EB virus. In our study, we do not present data regarding CMV infection nor the degree of CMV contamination in both study groups. In this regard, we would like to mention again the study by Bjornevik et al, who were able to convincingly show that CMV, in contrast to EBV, is not associated with an increased incidence of MS (4).

Although in our study the number of female MS patients clearly outweighs the number of male patients, a stronger association between EBV and MS is seen in male patients. This result, paradoxical at first sight, points to the important research field of gender-typical differences in possible risk factors and mechanisms in the development of autoimmune diseases (26, 27). Gender-specific influencing factors can be gene- and/or hormone-associated and can affect any aspect of an autoimmune response, including, as in our study, whether and how a viral infection is transfected into an autoimmune disease (28). In the development of MS after EBV infection, reinforcing signals seem to act in men, whereas in women other influencing factors might be more effective. Due to our study design, we cannot provide a definitive mechanistic explanation for the association between EBV and MS, but future mechanistic studies will certainly need to take gender-specific factors into account to unravel the complex interplay of the inflammatory pathways involved.

Our study is subject to limitations, which are unavoidable due to the database analysis and the study design. Firstly, we cannot exclude the possibility that diagnoses have sometimes been misclassified or that their coding is missing within the ICD-10 coding system. As such, analyses with respect to earlier disease stages of MS including the clinically isolated syndrome (CIS) were not feasible. In addition, there is a lack of data from specialized MS centres, which diagnose and treat a relatively big share of MS patients in Germany. Furthermore, the German Disease Analyzer Database does not contain information on patient lifestyle, risk factors like smoking or socioeconomic status as well as genetic factors or immunological markers, which are of clearly of high relevance to further dissect etiological aspects of MS. Likewise, data on disease severity or the subtype of MS were not available. We cannot provide data on mortality. As described above, our study is not based on serological determinations of an antibody titer against EBV, but on ICD-10 coding of a clinically apparent diagnosis of infectious mononucleosis. Therefore, it may well be that there is also an undetermined proportion of patients with EBV in the control group, which may dilute the differences between the two groups and may explain why the risk increase due to EBV in our study is lower than in the study by Bjorvik et al. (32-fold increased risk there). On the other hand, the very fact that the results in our study are nevertheless significant shows, that the association shown between EBV and MS is strong. In this context, we also believe that our main finding of a significantly increased risk of MS after clinical apparent mononucleosis at a young age is a very good complement to the new study by Bjornevik et al. (4).

In summary, we found a significantly increased incidence of MS in patients with clinically apparent EBV infection. Therefore, our study should lead to functional studies on a potential causal relationships between both entities and involved signalling pathways. These data could improve our understanding of the development of MS and possibly help to find novel therapies. As an example, future preventive options could include EBV vaccination for genetically vulnerable relatives of MS patients.

## Data availability statement

The raw data supporting the conclusion of this article will be made available upon reasonable request from the corresponding authors.

## Ethics statement

Ethical review and approval was not required for the study on human participants in accordance with the local legislation and institutional requirements. Written informed consent for participation was not required for this study in accordance with the national legislation and the institutional requirements.

## Author contributions

SL, KK, and CR designed the study. KK performed statistical analyses and generated figures and tables. SL, CD, CR, and KK wrote the manuscript. TL, SM provided intellectual input. All authors agreed to the final version of the manuscript. All authors contributed to the article and approved the submitted version.

## Funding

There was no specific funding for this study. In general, work in the group of TL was funded from the European Research Council (ERC) under the European Union’s Horizon 2020 research and innovation program through the ERC Consolidator Grant PhaseControl (Grant Agreement n° 771083). The lab of TL was further supported by the German Cancer Aid (Deutsche Krebshilfe 110043 and a Mildred-Scheel-Professorship) and the German-Research-Foundation (SFB-TRR57/P06, LU 1360/3-1, CRC1380/A01, and CA 830/3-1).

## Conflict of interest

Author KK was employed by IQVIA.

The remaining authors declare that the research was conducted in the absence of any commercial or financial relationships that could be construed as a potential conflict of interest.

## Publisher’s note

All claims expressed in this article are solely those of the authors and do not necessarily represent those of their affiliated organizations, or those of the publisher, the editors and the reviewers. Any product that may be evaluated in this article, or claim that may be made by its manufacturer, is not guaranteed or endorsed by the publisher.
